# Electrospun Graphene Oxide/Poly(m-phenylene isophthalamide) Composite Nanofiber Membranes for High Performance

**DOI:** 10.3390/membranes15050145

**Published:** 2025-05-12

**Authors:** Enling Tian, Yinping Bi, Yiwei Ren

**Affiliations:** 1 Chongqing Institute of Green and Intelligent Technology, Chinese Academy of Sciences, Chongqing 400714, China; 2 Chongqing School, University of Chinese Academy of Sciences, Chongqing 400714, China

**Keywords:** electrospinning, PMIA, graphene oxide, composite nanofiber membrane, air filtration

## Abstract

Due to its distinctive two-dimensional structure and high specific surface area, graphene oxide (GO) is expected to be a very promising material to be used for membrane separation. Not only can it improve the mechanical strength, surface wettability, and thermal stability of the membrane, but it can also improve the filtration performance and shelf life of the polymer membrane. Graphene oxide/poly(meta-phenylene isophthalamide) (GO/PMIA) nanofiber membranes were prepared by means of an electrospinning technique. The effects of adding different amounts of GO on the PMIA nanofiber membranes were studied. The results indicated that the GO had a strong affinity with the PMIA matrix by forming hydrogen bonds. The composite nanofiber membranes exhibited better filtration and thermostability performance than those of the pristine membrane. As the loading amount of GO was 1.0 wt%, the air filtration efficiency of the composite nanofiber membrane was 97.79%, the pressure drop was 85.45 Pa and the glass transition temperature was 299.8 °C.

## 1. Introduction

In recent years, the environmental characteristics of air pollution have become more complex [1,2,3,4,5,6,7]. Fine particulate matter (PM, i.e., the solid particles in the air) and ozone pollution are constantly aggravated while the emission levels of sulfur dioxide and nitrogen oxides lead to huge demands on the dust emissions from chemical industry, oil refineries, incinerations, metal refining and metal recycling. In particular, the notorious PM2.5 (with particle sizes ≤ 2.5 μm) can cause fatal lung carcinoma, which is classified as a first-level carcinogen by the World Health Organization (WHO) [8]. Moreover, it carries microorganisms involving bacteria and viruses, which can cause a major threat to public health, and is likely to lead to an outbreak of infectious diseases [9]. These fine particles can deeply penetrate human organs, such as the throat, the lungs and even the bloodstream system, thus causing detrimental diseases. However, most airborne PM generated at high temperatures (70–250 °C) primarily derive from sources such as industrial flue gas (140–200 °C), coal furnaces (70–200 °C) and automobile exhausts (70–80 °C) [10]. Hence, the development of air filter materials with high temperature resistance, corrosion resistance, high filtration efficiency and low wind resistance performance is imperative.

Hot gas clean-up technology is a technology that reduces the concentration of dust or removes dust from the gas under high-temperature conditions [11]. At present, the main process of treating hot gas includes cooling and filtering, where the temperature of the hot gas is reduced to about 200 °C~250 °C by the cooling measures. High-temperature filter materials generally refer to filter materials that can still exhibit favorable performance above 200 °C. A bag filter can be used at high temperatures. Traditional high-temperature inorganic fiber filters, such as ceramic fiber, high silica glass fiber, basalt fiber, metal fiber and glass fiber, are usually used in bag filter technology. However, such filtration materials present disadvantages such as thick fiber diameter, large pore size, and low filtration efficiency, which would not meet the basic need of high-temperature resistant filtration. Fortunately, electrospun nanofibers present significant advantages in view of their high aspect ratios, small fiber diameter and thickness, high porosity, and large specific surface area [12,13], allowing them to effectively intercept fine PMs. Whereas high-temperature organic fiber filters mainly include polyimide (PI) fiber [14], polyphenylene sulfide (PPS) fiber [15], aramid, polytetrafluoroethylene (PTFE) fiber [16], aromatic sulfone fiber, poly(meta-phenylene isophthalamide) (PMIA) fiber and so on. In comparison, PMIA exhibits excellent thermal resistance, is flame retardant and has superior mechanical properties, and is being widely utilized as the base material for high-temperature resistant filters [17]. In practical engineering applications, in order to improve the overall performance of the filter, fibers are often elected to be modified or produced with different processes.

Fiber modification refers to the physical and chemical treatment of the fiber, thereby improving the performance of the fiber filter. At present, there are several methods to modify the fiber, including surface treatment and blending modification. Fiber surface treatment includes surface oxidation, surface coating, surface grafting, ray irradiation and so on [18,19]. Blending modification involves mixing two or more substances together. In these modification methods, blending the nano-particles and polymer matrix presents many advantages, such as integrating the mechanical properties, electrical properties and thermal performance of the nano-particles into the blends on a nanoscale. Without improving the performances of the polymer matrix such as machinability, stability, weight and other characteristics, the performance of the composite materials on the macroscale will be greatly improved [20,21]. Nano-materials usually include carbon nanotubes [22], nano-silica [23], titanium dioxide [24], nano-clay [25], graphene [26] and so on [27,28]. Among them, graphene oxide (GO) is a derivative of graphene, with the features of good hydrophilicity, high specific surface energy and good mechanical properties. The layer spacing of GO is 6~10 Å and the rich oxygen-containing functional groups enable it to disperse stably in the vast majority of polar organic solvents [29]. Therefore, it could blend with a variety of polymer matrices.

In this paper, GO was mixed with PMIA, and the GO/PMIA composite nanofiber membrane was prepared using electrospinning technology. The thermal stability and air filtration performance of the PMIA nanofiber membrane were improved by doping GO. The effects of different contents of GO on the properties of the GO/PMIA nanofiber membranes were investigated. It was found that hydrogen bonds were formed between the GO and the PMIA molecules. As the amount of GO was 1.0 wt%, the diameter of the composite nanofiber membrane showed a narrow size distribution, and the hydrophilicity, thermal stability and air filtration performance of the composite nanofiber membranes were also improved. 

## 2. Experimental Section and Methods

### 2.1. Materials and Chemicals

The materials and chemical used in this study include the commercialized poly(meta-phenylene isophthalamide) (PMIA, M_w_ = 200,000, Yantai Tayho Advanced Materials Co., Ltd., Yantai, China) short fibers, N,N-dimethylacetamide (DMAc, Kaloon Chemical Industry Co., Ltd., China), Lithium chloride anhydrous (LiCl, Kaloon Chemical Industry Co., Ltd. China), graphene oxide (GO, Nanjing XFNANO Materials Tech Co., Ltd., Nanjing, China, XF002-02). All of the materials and chemicals were used without further purification.

### 2.2. Preparation of Spinning Solution

The GO/PMIA solution for electrospinning was prepared as follows. Firstly, GO powder was dissolved in DMAc with the additive of LiCl at room temperature under constant stirring for 24 h and consequently ultrasonicated for 90 min (JY98-IIIN Shanghai Jingxin Industrial Development Co., Ltd., Shanghai, China). The power of ultrasonication was 1200 W at 25% efficiency. Then, a calculated amount of PMIA short fibers were dissolved in the above solution and stirred at 80 °C for 24 h to ensure homogeneity. Finally, GO in PMIA with different contents of 0.0 wt%, 0.4 wt%, 1.0 wt%, 2.0 wt% and 3.0 wt% were finished. The composition of polymer and additive is illustrated in Table 1.

### 2.3. Preparation of GO/PMIA Composite Nanofiber Membranes

The GO/PMIA mixed solution was processed by applying an electrospinning system (Shanghai Yuyue Nano Technologies Co., Ltd., Shanghai, China) consisting of a 10 mL syringe which was driven by a syringe pump. The length and the inner diameter of the needle were 30 mm and 0.51 mm, respectively. The solution flow rate was fixed at 0.1 mL·h^−1^. Before electrospinning, a metallic silicon paper (30 cm × 70 cm) was mounted on the fiber collecting drum (21 cm in diameter) and with a rotation rate of 30 rpm. The electrospinning was conducted at the applied positive voltage of 25 kV with a working distance of 20 cm under ambient conditions (relative humidity 55%~65%, at room temperature). Then, the GO/PMIA composite nanofiber membrane was prepared. The thickness of nanofiber membranes was controlled by the spinning time. The resultant electrospun composite nanofiber membranes were denoted as PG0, PG1, PG2, PG3 and PG4, corresponding to the addition of GO content 0.0 wt%, 0.4 wt%, 1.0 wt%, 2.0 wt% and 3.0 wt%, respectively.

### 2.4. Characterization of Composite Nanofiber Membranes

The Raman spectra were recorded on a Laser confocal Raman microscope (inVia Reflex, Renishaw, Gloucestershire, UK) using an excitation wavelength of 785 nm. The morphology of composite nanofiber membranes was observed by field emission scanning electron microscopy (FE-SEM). The SEM samples were sputter-coated with platinum for 60 s to prevent charging during SEM imaging. The diameters of the fibers in the SEM images were measured using image analysis software of the instrument. The crystallinity of composite nanofiber was characterized by X-ray diffraction (XRD, X’ Pert 3 Powder, PANalytical, Almelo, The Netherlands), with Cu-Kɑ (λ = 1.54 Å) as the excitation source. The scanning range was 2θ = 5°~45°, and the scanning rate was 0.02 s^−1^. A dynamic contact angle measuring instrument (DSA 100, KRÜSS, Hamburg, Germany) was utilized to characterize the hydrophilicity/hydrophobicity of the composite nanofiber. A total of 3 μL deionized water droplets were placed on the surface of the fiber film, and 8 random points were measured for each sample. The filtration efficiency and air flow resistance were measured by an automated filter tester (TSI-8130) [30]. The tester could deliver charge-neutralized monodisperse solid NaCl aerosol particles that had a mass median aerodynamic diameter of 0.26 mm and count median diameter of 0.075 μm with geometric standard deviation not exceeding 1.83. All NaCl aerosol tests were conducted at room temperature with a continuous airflow rate of 32 L·min^−1^. Thermogravimetric analysis (TGA) was investigated using a simultaneous thermal analyzer (TGA/DSC1, Mettler Toledo, Shanghai, China) with the temperature increasing from 50 °C to 800 °C at a heating rate of 10 °C·min^−1^ under a 50.0 mL·min^−1^ nitrogen atmosphere. The melting behaviors of the samples were tested by differential scanning calorimetry (DSC) using a DSC analyzer (DSC1, Mettler Toledo, Shanghai, China) under nitrogen atmosphere. The samples were first heated to 370 °C (to remove any previous processing history) from 25 °C, then cooled to 150 °C, and finally heated to 500 °C. The heating and cooling rates for all runs were 10 °C·min^−1^. 

## 3. Results and Discussion

### 3.1. Structure of PMIA/GO Composite Nanofiber Membrane

**Figure 1:**
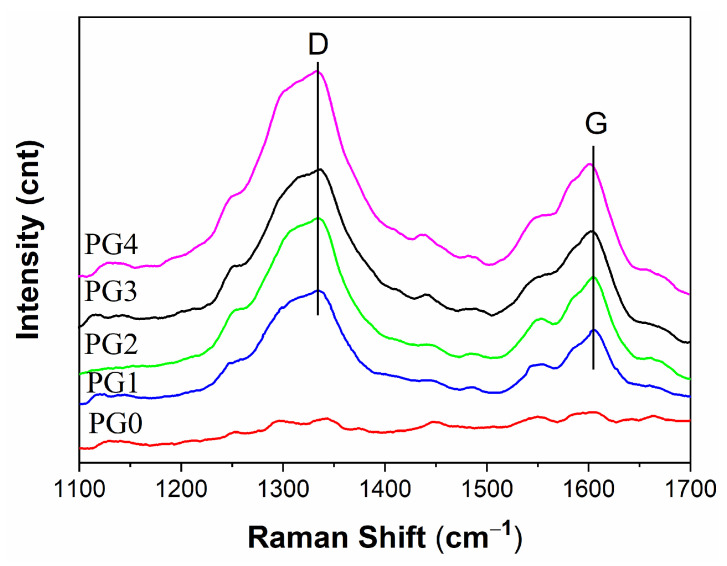
Raman spectra of different GO weight ratios of GO/PMIA composite nanofiber membranes.

Raman spectroscopy is usually used to analyze the structure of carbon nanotubes, graphene and its derivatives. Figure 1 shows the Raman spectra of GO/PMIA composite nanofiber membranes. It can be seen that there were two characteristic bands: the D band at ~1335 cm^−1^ and the G band at ~1600 cm^−1^. The D band showed the carbon material defects and irregularities. The G band represented the characteristics of carbon sp^2^, which reflected the symmetry and regularity of the carbon material. The highly ordered graphite D-band (1355 cm^−1^) was caused by defects in the graphite, and the G-band (1575 cm^−1^) was the same phase vibration of the graphite lattice [31]. GO is the oxidation product of graphite, and band D and band G were widened. As the composite nanofiber membranes varied from PG1 to PG4, the calculated area ratio between D and G bands was 2.4528, 2.5685, 2.8946, 3.1461, respectively, according to the multi-modal fitting. Accordingly, the intensity of the D band became larger and the G band moved to the large wave number. The pure PMIA nanofiber membrane (PG0) did not exhibit a distinct D band and G band, while the composite nanofiber membranes did. Therefore, GO was successfully doped into the PMIA fiber membrane.

### 3.2. Morphology of PMIA/GO Composite Nanofiber Membrane

**Figure 2:**
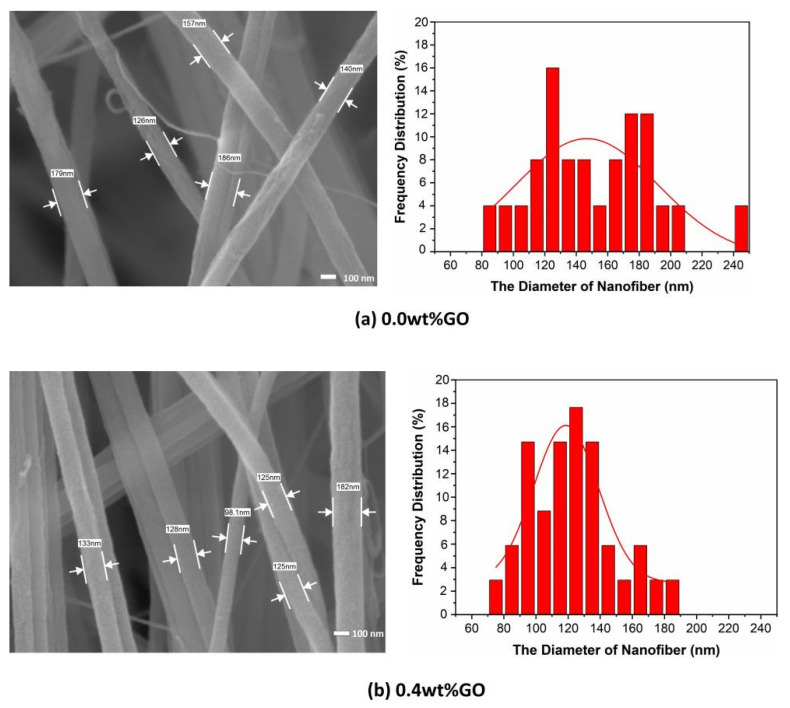

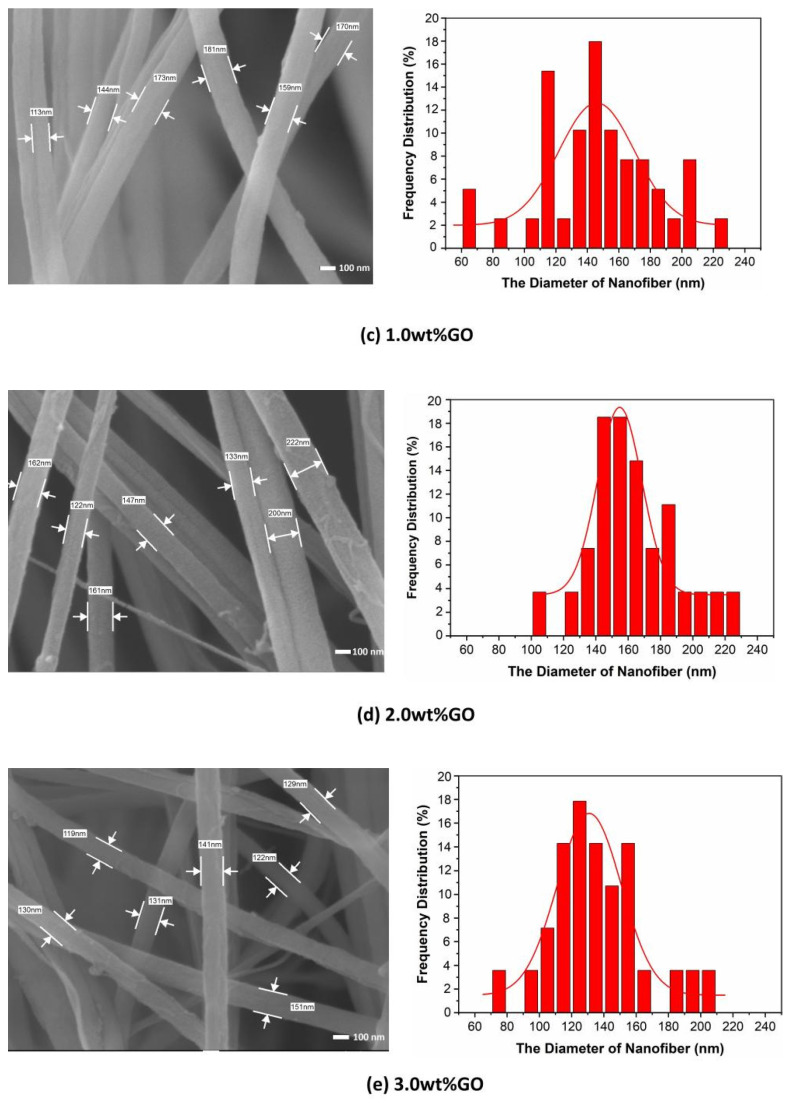
The FE-SEM images and the diameter distributions of GO/PMIA composite nanofiber membranes.

Figure 2 shows the FE-SEM images and the fiber diameter distribution of GO/PMIA composite nanofiber membranes prepared by electrospinning. As can be seen from Figure 2, the fibers were randomly stacked together, with alternating thickness, and layers spread out, with a three-dimensional network structure. Some fibers bonded together and presented a certain degree of orientation, which was the result of moisture phase separation and charge dissipation processes [32]. After measuring the diameter of 150 nanofibers and calculating the diameter distribution, it can be seen from the distribution diagram that the distribution of nanofibers diameter changed with the increase in GO content. The diameter distribution of the nanofibers with the GO addition was narrower than that of the pure PMIA nanofibers.

**Figure 3:**
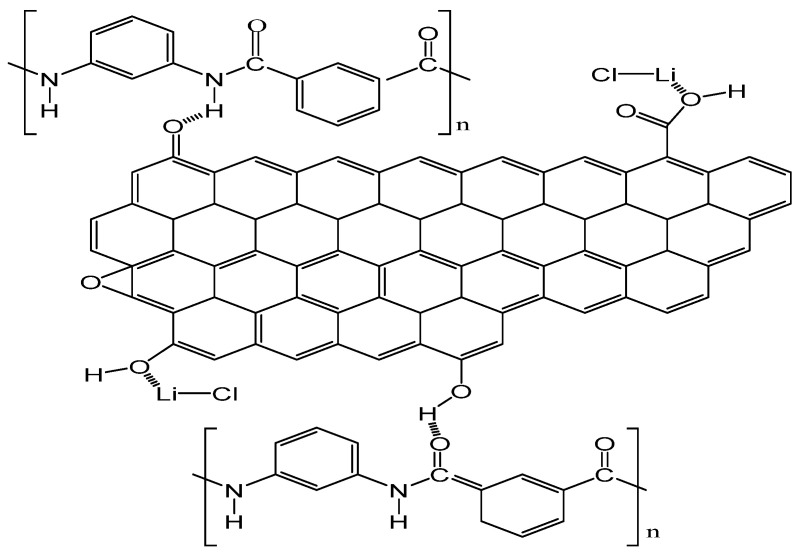
Schematic representation of PMIA and LiCl with GO interactions.

The reason for the change in nanofiber diameter distribution may be that the hydrogen bond has formed between the oxygen-containing functional group of GO and the amide bond of PMIA. Furthermore, the interaction occurred between the metal ions of LiCl and the oxygen of GO (Figure 3 shows the interaction between PMIA and GO and LiCl and GO) [33]. The formation of hydrogen bonds increased the entanglement among the PMIA molecules and reduced the free movement of the PMIA molecular chains. Moreover, the interaction between LiCl and GO affected the nature of the electrospinning solution and ultimately led to differences in the nanofiber diameter distributions [34]. As the addition amount of GO was 3.0 wt%, the distribution of the nanofiber diameters was similar to that of the GO addition amount of 0.4 wt%. The reason may be that the degree of stripping of the GO nanosheets was different under the same power and the time of ultrasonic operation. Moreover, the GO nanosheets were prone to agglomerating when the content increased.

The porosity of all the electrospun nanofiber membranes was calculated using the following Equation (1) [35]:

(1) $$\mathrm{Porosity}=(1-\frac{m}{t\times A\times \rho })\times 100\%$$

where *m*, *t* and *A* are the mass, thickness, and per unit measured membrane, respectively. And *ρ* is the density of polymer raw material.

**Figure 4:**
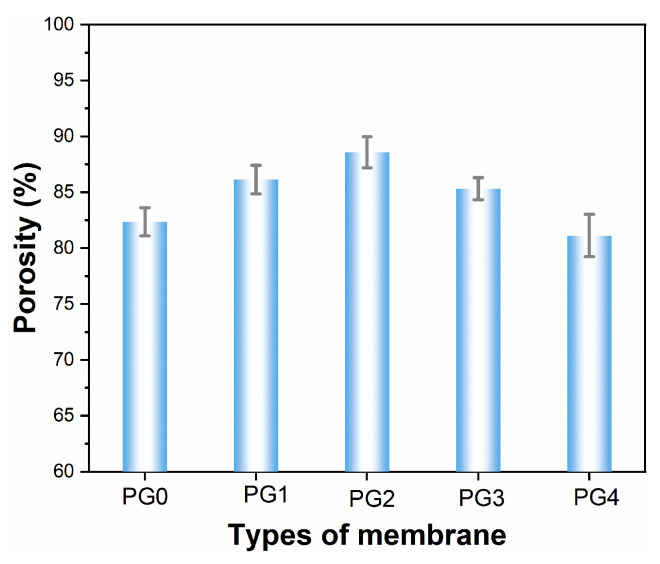
The effect of GO content on the porosity of composite nanofiber membranes.

The porosity of the electrospun nanofiber membranes was calculated according to Equation (1), which indicated that PG2 possessed the highest porosity, up to 88.58% (Figure 4). It is worth noting that the porosity of PG4 was lowest with the greatest addition of GO in the spinning solution. This phenomenon could be attributed to the agglomeration of the GO nanoparticles to some extent.

### 3.3. Crystallinity of PMIA/GO Composite Nanofiber Membrane

**Figure 5:**
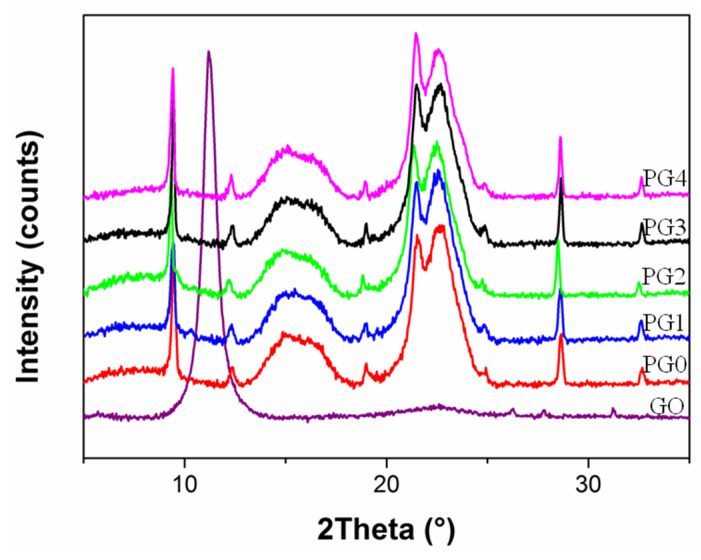
XRD spectra of PMIA/GO composite nanofiber membrane.

Figure 5 shows the XRD pattern of the PMIA/GO composite nanofiber membrane prepared by electrospinning. It can be seen from Figure 5 that GO has a characteristic diffraction peak at ~11°. It presented similar characteristic diffraction peaks in all the PMIA/GO composite nanofiber membranes. However, no characteristic peaks of GO appeared. Therefore, it can be deduced that the addition of GO had no effect on the crystal shape of PMIA.

### 3.4. Wettability of PMIA/GO Composite Nanofiber Membrane

**Figure 6:**
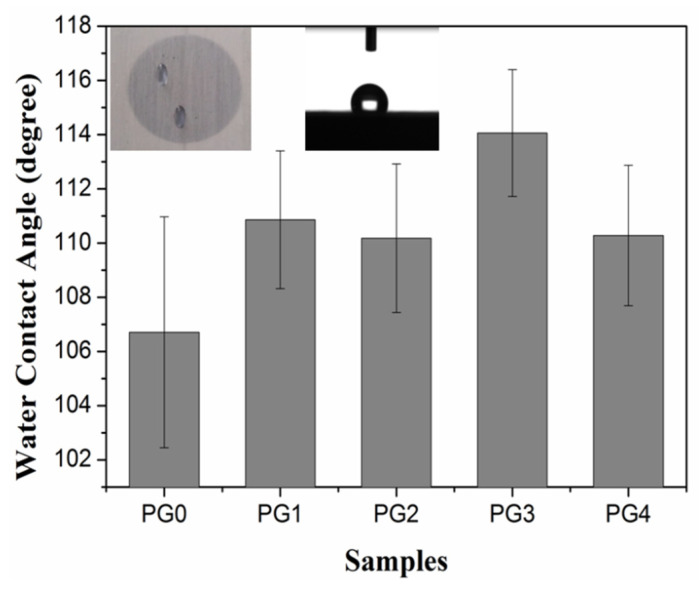
Contact angle of PMIA/GO composite nanofiber membrane.

The contact angle is the angle between the gas–liquid interface and the solid–liquid interface at the junction of gas, liquid and solid. Because there are plenty of pores among the nanofibers, the contact angle will be affected by many factors, such as the contact area between the water droplet and the fiber [36], as well as the roughness of the nanofiber surface [37]. Therefore, the interaction process between the fibers and the water is relatively complicated. It includes the wetting of the fiber surface, liquid flow caused by capillary action, absorption of water on the fiber surface and diffusion of water into the fiber interior, and so on. Figure 6 shows the contact angle of the PMIA/GO composite nanofiber film prepared by electrospinning. It can be seen from Figure 6 that all contact angles were greater than 90° as the droplets initially contacted the fiber membrane. Therefore, all fiber samples exhibited hydrophobic behavior, which may be due to the nano size effect of the nanofibers prepared by electrospinning. The contact angle varied with the increase in GO addition, possibly because the addition of GO affected the diameter distribution of nanofibers. It then affected the size of pores and further affected the contact area between water droplets and nanofibers. It was interesting to find that the water droplets permeated in the direction of the fiber orientation over time during the test.

### 3.5. Air Filtration Performance of PMIA/GO Composite Nanofiber Membrane

**Figure 7:**
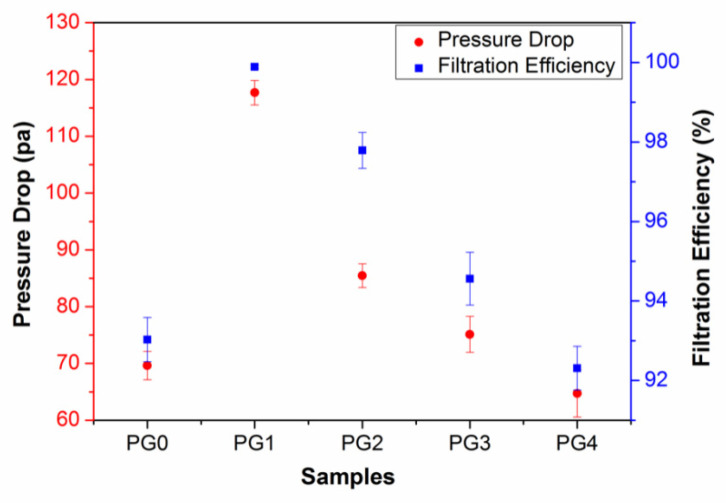
Filtration efficiency and pressure drop of GO/PMIA composite nanofiber membranes.

The effect of GO on the nanofiber diameter distribution indirectly affected the air filtration performance of the composite nanofiber membranes. On one hand, as the additional amount of GO was less than 0.4 wt%, the doping of GO led to the diameter distribution of nanofibers being narrower. Hence, the path of gas filtration became more tortuous [38], which improved the filtration efficiency. On the other hand, the size of the delaminated graphene in the composite nanofiber membrane increased with an increase in the amount of GO. And it was easy for GO to agglomerate as the GO content was high, thus causing the composite nanofiber structure to be loose and the filtration efficiency to decrease. Figure 7 shows the filtration efficiency and the pressure drop of GO/PMIA composite nanofiber membranes. It can be seen from Figure 7 that the filtration efficiency increased first and then decreased with the increase in GO content. The filtration efficiency of PG1 was 98.99% and the filtration resistance was 117.68 Pa, which was the maximum. The pressure drop also exhibited a similar trend. Although the air filtration efficiency of the composite nanofiber membrane improved, the resistance increased with the GO content increasing. And a good air filtration membrane is needed to have both high filtration efficiency and low air resistance. Therefore, the mass factor (QF) was introduced to evaluate the air filtration performance of composite nanofiber membranes using Equation (2) [35]:

(2) $$QF=-\frac{\ln(1-\eta )}{\Delta P}$$

where *η* was air filtration efficiency and Δ*P* represented air resistance. Thereby, calculated by Equation (2), the mass factor QF of PG0, PG1, PG2, PG3, PG4 was 0.038, 0.039, 0.045, 0.039 and 0.040, respectively. Therefore, in the practical application, PG2 was more economical.

### 3.6. Thermal Performance of PMIA/GO Composite Nanofiber Membrane

**Figure 8:**
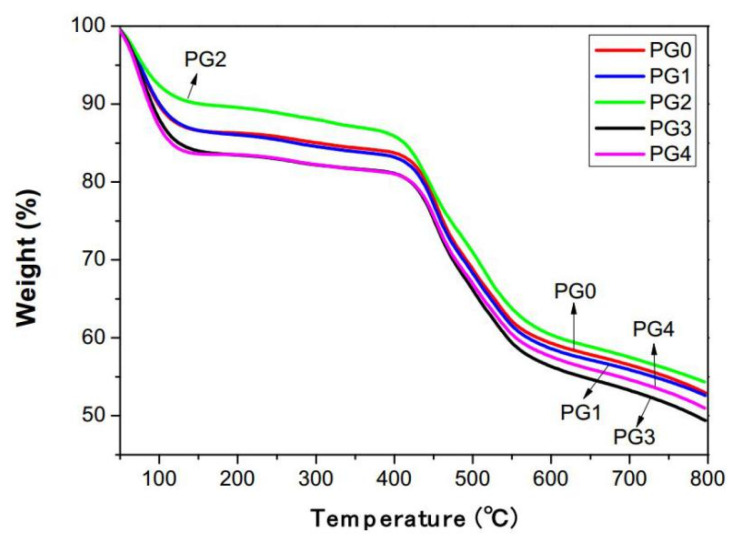
The TGA curves of GO/PMIA composite nanofiber membranes.

Figure 8 presents the thermal weight loss curves of the GO/PMIA composite nanofiber membranes. As can be seen from Figure 8, the composite nanofiber membranes exhibited four weight loss stages as the temperature increased. The weight loss in the range of 25~150 °C in the first stage may be due to the volatilization of the adsorbed water of the composite nanofiber membranes. The mass loss in the second stage in the range of 200~400 °C was the result of the hydrogen bond breaks and thermal decomposition of GO to produce water molecules. And the weight loss in the range of 400~500 °C in the third stage was caused by the hydrolysis and homolysis of PMIA into small molecules such as H_2_O, HCN and aniline [39]. The fourth stage was above 500 °C and aromatic hydrocarbon began to dehydrogenate [40,41]. As the addition amount of GO was 0.4 wt%, the thermal weight loss curve of the composite nanofiber membrane was almost the same as that of the pristine PMIA nanofiber membrane. As the addition amount of GO was 1.0 wt%, the mass loss of the composite nanofiber membrane was minimal and the thermal stability optimal. As the GO content continued to increase, the mass loss rate of the composite nanofiber membrane increased and the thermal stability decreased. The reason may be that the crystallization of PMIA was incomplete. The recrystallization occurred as the temperature elevated and the small amount of GO played the effect of nucleation, so as to improve the thermal stability of the composite nanofiber membrane. As the GO content was in excess of 1.0 wt%; GO itself decomposed at 200 °C. In addition, due to the existence of hydrogen bonds between GO and PMIA molecules, and between internal PMIA molecules being destroyed, the thermal stability of the composite nanofiber membranes is reduced.

**Figure 9:**
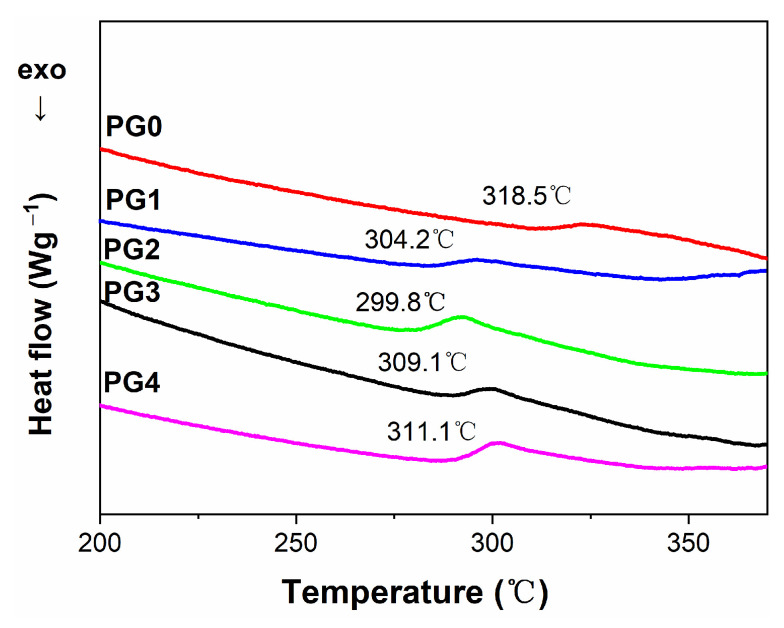
The DSC curves of GO/PMIA composite nanofiber membranes.

Figure 9 illustrates the DSC spectra of GO/PMIA composite nanofiber membranes. It can be seen from Figure 9 that the glass transition temperature (Tg) of GO/PMIA composite membranes decreased first and then increased with the increase in GO content. As the addition of GO was 1.0 wt%, the Tg was 299.8 °C. As the content of GO was more than 1.0 wt%, the Tg increased with the increase in GO content; however, it was still lower than that of the pristine PMIA membrane. The reason for this phenomenon was that the lamellar spacing of GO was less than the distance required for the relaxation of the molecular chains of the polymer, and the PMIA molecules inserted between the lamellae were rapidly relaxed, resulting in a decrease in Tg ([28,42]). The flexibility of the composite nanofiber membranes was relatively increasing with Tg decreasing. The relaxation depended not only on the degree of dispersion of GO nanosheets in the PMIA matrix but also on the interaction between GO and PMIA molecules [43,44]. The presence of a hydrogen -bond between GO and PMIA molecules led the original hydrogen bond network in PMIA intermolecular to be destroyed [45], thus reducing the stability of PMIA molecules. The Tg increased with the increase in GO addition in that the GO nanosheets were prone to agglomerate and the degree of dispersion and the interaction woke. In addition, the endothermic peak at 440 °C was the endothermic peak of PMIA decomposition.

## 4. Conclusions

The GO/PMIA composite nanofiber membranes were successfully fabricated by electrospinning. The aforementioned analysis showed that GO has good miscibility with the PMIA matrix. Although the Tg of the nanocomposite membranes was lower than the pristine membrane, the GO/PMIA nanofiber membranes still exhibited many desirable properties, including spinnability, thermostability and preferable filtration performance. Meanwhile, the hydrophobicity of the composite nanofiber membranes was also improved. As the additive amount of GO nanosheets reached 1.0 wt%, the PMIA matrix had a strong affinity with GO nanosheets by forming hydrogen bonds. And the GO nanosheets were fully exfoliated and uniformly dispersed into the interstitial void of the PMIA crystal lattice. Moreover, much more torturous paths were constructed by adding the GO sheets. Benefiting from the enhanced properties of GO/PMIA membranes, the nanofiber membranes exhibited better filtration efficiency than the pristine membrane when tested with 300–500 nm sodium chloride aerosol particles. Consequently, GO is a desirable reinforcing filler to improve the filtration performance of the PMIA nanofiber membranes.

## Figures and Tables

**Table 1:** Polymer and additive composition.

Membrane Code.	PMIA *^ɑ^* (wt%)	DMAc *^ɑ^* (wt%)	LiCl *^ɑ^* (wt%)	GO *^β^* (wt%)
PG0	10	88	2	0
PG1	9.9960	87.9648	1.9992	0.4
PG2	9.9900	87.9121	1.9980	1.0
PG3	9.9800	87.8244	1.9960	2.0
PG4	9.9701	87.7368	1.9940	3.0

Note: “*ɑ*” represents the percentage of a component mass in the total mass; “*β*” represents the percentage of GO mass in the PMIA mass.

## Data Availability

The data presented in this study are available on request from the corresponding author due to privacy, legal or ethical reasons.
